# The Emerging Role of N-Lactoyl-Phenylalanine (Lac-Phe) in Metabolic Regulation and Disease: From Exercise-Induced Metabolite to Therapeutic Candidate

**DOI:** 10.3390/antiox15040441

**Published:** 2026-04-01

**Authors:** Julia Chu-Ning Hsu, Chia-Hui Chen, Ming-Wei Chen, Wen-Hua Chen, Tzong-Shyuan Lee

**Affiliations:** 1 Department of Veterinary Medicine, College of Veterinary Medicine, National Chung Hsing University, 145, Xingda Road, South District, Taichung 402202, Taiwan; juliacnhsu@dragon.nchu.edu.tw; 2 Graduate Institute and Department of Physiology, College of Medicine, National Taiwan University, 1, Jenai Road Section 1, Zhongzheng District, Taipei 100233, Taiwan; chiahuichen1993@ntu.edu.tw (C.-H.C.); brian0512@nari.org.tw (M.-W.C.); f08441005@ntu.edu.tw (W.-H.C.); 3 Department of Isotope Application Research, National Atomic Research Institute, 1000, Wenhua Road, Longtan District, Taoyuan 325207, Taiwan

**Keywords:** N-lactoyl-phenylalanine, exerkine, appetite suppression, obesity, mitochondrial dysfunction, metformin, redox homeostasis, oxidative stress

## Abstract

N-Lactoyl-phenylalanine (Lac-Phe), identified in 2022 as an exercise-inducible signaling metabolite, is formed by carnosine dipeptidase 2 via conjugation of lactate and phenylalanine. Its circulating levels rise sharply after intense exercise in mice, humans, and racehorses, reflecting increased glycolytic flux. Beyond exercise, Lac-Phe also rises with feeding and metformin, positioning it as a potential integrator of energy intake, expenditure, and metabolic homeostasis. Centrally, Lac-Phe may contribute to appetite suppression by inhibiting hypothalamic orexigenic agouti-related protein neurons, primarily observed in obese rodent models, while sparing anorexigenic pro-opiomelanocortin neurons, thereby reducing food intake, promoting weight loss, and improving glucose tolerance in obese models without altering energy expenditure. Peripherally, it drives anti-inflammatory M2 macrophage polarization, ameliorating colitis and aiding recovery after spinal cord injury via NF-κB suppression and reactive oxygen species reduction. As a biomarker, Lac-Phe may offer advantages over lactate in reflecting mitochondrial dysfunction in conditions such as MELAS, sepsis, and NADH-reductive stress; however, these observations derive mainly from small-scale or exploratory studies and require prospective validation. Recent studies from 2024 to 2025 further reveal its partial and context-dependent role in mediating metformin’s effects, intensity- and sex-dependent responses, renal clearance via SLC17A1/3 transporters, and links to exercise-induced redox adaptations. The first human phase I trial (NCT06743009), launched in 2025, is assessing the metabolic effects of Lac-Phe in obesity. This Perspective summarizes Lac-Phe biosynthesis, physiological mechanisms, including its emerging but largely correlative connections to redox homeostasis, and therapeutic promise, underscoring its potential relevance for exercise-mimicking strategies in metabolic, inflammatory, and redox-related disorders.

## 1. Introduction

In the rapidly evolving field of metabolic biology, the discovery of signaling metabolites that bridge cellular processes with systemic physiology has opened new avenues for understanding health and disease. One such molecule, N-lactoyl-phenylalanine (Lac-Phe), has garnered significant attention since its identification in 2022 as an exercise-inducible metabolite [1]. Lac-Phe, a conjugate of lactate and phenylalanine, exemplifies the concept of “exerkines,” which are molecules released in response to physical activity that mediate its physiological benefits [1,2,3]. Initially recognized for its role in appetite suppression and obesity reduction, the biological footprint of Lac-Phe has since expanded to encompass metabolic regulation, modulation of inflammation, and potential use as a biomarker for mitochondrial disorders [2,3,4,5]. Lac-Phe was first identified through untargeted metabolomics studies in exercised animals and humans, which revealed marked elevations in its circulating levels following physical activity [1,2,3]. In mice, racehorses, and humans, Lac-Phe levels surge following intense exercise and correlate with lactate production from glycolysis [1]. This metabolite is synthesized via reverse proteolysis catalyzed by carnosine dipeptidase 2 (CNDP2), an enzyme ubiquitously expressed in tissues such as macrophages, monocytes, and epithelial cells [1,3,6]. Beyond exercise, Lac-Phe is also elevated by feeding and by pharmacological agents such as metformin, positioning it as a central mediator linking energy intake, energy expenditure, and metabolic homeostasis [1,3,5,7].

Biomedical interest in Lac-Phe arises from its multifaceted biological roles. In preclinical models, it suppresses food intake without altering energy expenditure, leading to weight loss and improved glucose tolerance [1,3,7]. Clinically, circulating Lac-Phe levels correlate with metabolic health, and ongoing clinical trials initiated in 2025 are evaluating its therapeutic potential for obesity [8]. Moreover, Lac-Phe and related N-lactoyl-amino acids have emerged as biomarkers for conditions involving mitochondrial dysfunction, including septic shock and mitochondrial encephalopathy with lactic acidosis and stroke-like episodes (MELAS) syndrome [1,3,5,9,10,11,12]. These metabolites have shown preliminary potential as biomarkers that may complement or outperform traditional markers such as lactate, though larger prospective studies are needed to confirm their clinical utility [1,11,12]. This mini-review explores the biosynthesis, physiological functions, therapeutic implications, and future research directions of Lac-Phe, highlighting its transformative potential in biomedical science.

## 2. Biosynthesis and Regulation of Lac-Phe

The biosynthesis of Lac-Phe represents a fascinating convergence of glycolytic flux and amino acid metabolism [3]. The molecule is formed by conjugating lactate, a byproduct of anaerobic metabolism, with phenylalanine, an essential amino acid [1,12,13,14]. Structurally, Lac-Phe is an N-acyl amino acid in which the carboxyl group of L-lactate forms an amide bond with the alpha-amino group of L-phenylalanine, yielding a dipeptide-like conjugate that is chemically distinct from both its precursors and from classic peptide hormones [1,6]. This reaction is facilitated by CNDP2, a cytosolic enzyme traditionally characterized as a dipeptidase but also capable of mediating the reverse condensation reaction under conditions of elevated substrate availability [1,6]. Under physiological conditions, CNDP2 normally hydrolyzes dipeptides; however, when intracellular concentrations of lactate and phenylalanine rise sufficiently, the thermodynamic equilibrium shifts to favor the condensation (ligation) reaction, thereby driving Lac-Phe synthesis in a substrate-driven, enzyme-catalyzed manner without requiring additional energy input or transcriptional regulation [1,6]. This mass-action mechanism makes Lac-Phe production exquisitely sensitive to cellular metabolic state rather than to hormonal signaling cascades, conferring rapid, tissue-level responsiveness to changes in energy metabolism [3,6]. CNDP2 expression is widespread, occurring in immune cells such as macrophages and monocytes, as well as in epithelial tissues and various other organs [1]. This broad distribution permits decentralized production of Lac-Phe, in contrast to the centralized secretion characteristic of classical hormones [1,2,15]. Regulation of Lac-Phe is tightly linked to metabolic flux, with important modulators including exercise intensity (high-intensity bouts elicit greater responses), type (e.g., sprint vs. endurance), volume, and nutritional status, although these parameters remain incompletely characterized, especially in humans [1,2,15]. Exercise, particularly high-intensity bouts, enhances glycolytic throughput, increasing lactate availability and thereby fueling Lac-Phe synthesis [1,3,6,14]. During intense exercise, skeletal muscle operates beyond its oxidative capacity, leading to accelerated glycolysis and a marked rise in intracellular and circulating lactate. This lactate surplus, combined with phenylalanine released from protein turnover and dietary sources, floods CNDP2-expressing tissues, particularly macrophages and gut epithelial cells, providing both substrates necessary for the reverse-proteolysis reaction and thereby driving a rapid, proportional increase in Lac-Phe synthesis [1,3,6]. Studies in mice have demonstrated that plasma Lac-Phe concentrations peak immediately after running and remain elevated for several hours thereafter [1]. Similarly, in humans, treadmill exercise can increase circulating Lac-Phe levels by up to threefold, with responses varying by exercise type, such as sprinting versus endurance training [1,2]. This intensity dependence reflects the substrate-driven nature of CNDP2 catalysis: higher exercise intensities generate proportionally greater lactate, which in turn augments Lac-Phe output, making circulating Lac-Phe a real-time reporter of glycolytic activity [1,2]. Feeding also stimulates Lac-Phe production. Following a meal, the postprandial rise in circulating phenylalanine, derived from dietary protein digestion, serves as the primary driver, combining with basal lactate levels already present in tissues to increase Lac-Phe biosynthesis. This amino acid-driven pathway is distinct from the glycolysis-driven increase observed during exercise [5,15]. Following a meal, the absorptive rise in circulating phenylalanine, derived from dietary protein digestion, acts as the rate-limiting substrate, as basal lactate is already present in most tissues. The resultant surge in Lac-Phe after feeding thus reflects a distinct physiological arm of biosynthetic regulation that is amino acid-driven rather than glycolysis-driven, suggesting that CNDP2 integrates signals from both energy metabolism and protein nutrition [5,15]. Pharmacologically, metformin increases circulating Lac-Phe levels in both humans and rodents by inhibiting mitochondrial complex I, particularly in intestinal epithelial cells, thereby elevating intracellular lactate that fuels CNDP2-mediated Lac-Phe synthesis [5,7,16]. This pathway partially accounts for the appetite-suppressing and weight-reducing effects of metformin observed in rodent models, as these benefits are significantly attenuated in CNDP2-knockout animals [7]. In humans, metformin treatment has been consistently associated with elevated Lac-Phe levels across multiple observational and interventional studies, although the extent to which Lac-Phe contributes to its clinical metabolic benefits remains to be fully determined [5].

In pathological states, Lac-Phe regulation shifts markedly. In mitochondrial disorders such as MELAS (mitochondrial encephalomyopathy, lactic acidosis, and stroke-like episodes), circulating Lac-Phe levels are elevated, likely due to NADH-reductive stress and impaired oxidative phosphorylation, resulting in lactate accumulation [1,3,5,10,12]. Similarly, in septic shock, Lac-Phe and related N-lactoyl-amino acids rise substantially and have been associated with mitochondrial dysfunction and disease severity [11]. In phenylketonuria (PKU), a genetic disorder characterized by excessive phenylalanine accumulation, Lac-Phe levels increase as a spillover product of elevated substrate availability [6,17]. However, evidence in these pathological contexts is predominantly derived from small human cohort studies or exploratory metabolomics analyses, with limited mechanistic validation in larger prospective trials. Systemic homeostasis of Lac-Phe is maintained through renal excretion, which is mediated by the kidney-restricted solute carrier transporters SLC17A1 and SLC17A3 in both mice and humans [18]. Genetic determinants, including polymorphisms in the CNDP2 gene and potentially in SLC17 transporters, can influence baseline Lac-Phe concentrations and responsiveness to physiological or pathological stimuli [1,6,18]. Genetic determinants, including polymorphisms in the CNDP2 gene and potentially in SLC17 transporters, can influence baseline Lac-Phe concentrations and responsiveness to physiological or pathological stimuli [7]. Collectively, these findings highlight the dynamic regulation of Lac-Phe in response to both physiological and pathological cues, underscoring its potential as a versatile therapeutic target.

## 3. Physiological Function and Mechanisms of Lac-Phe

The physiological functions of Lac-Phe have been studied predominantly in rodent models and remain to be fully validated in humans [1,3,14,19]. These actions can be organized into central neuroendocrine regulation, peripheral immune modulation, and systemic metabolic effects. At the central level, Lac-Phe may act as an anorexigenic signal by suppressing orexigenic agouti-related protein (AgRP) neurons in the hypothalamus while preserving anorexigenic pro-opiomelanocortin neuronal activity. The cognate receptor for Lac-Phe on hypothalamic neurons has not yet been identified, and the precise downstream signaling cascade (including the reported involvement of ATP-sensitive potassium channels) remains hypothetical [4]. This selective inhibition reduces food intake in diet-induced obese mice without affecting locomotor activity or basal metabolic rate, as evidenced by unchanged oxygen consumption and respiratory exchange ratios [1,7]. In diet-induced obese mice, chronic intraperitoneal administration of Lac-Phe (50 mg/kg) reduces body weight by 10–15% over several weeks, with decreased adiposity and improved insulin sensitivity [1].

To contextualize Lac-Phe within classical appetite regulation, hormones such as leptin (long-term energy store signaling) and ghrelin (hunger promotion) play central roles. Lac-Phe appears to acutely suppress feeding and can do so even in leptin-resistant, obese states; however, potential interactions with ghrelin signaling have not been examined. Peripherally, Lac-Phe has been shown in murine models to promote M2 macrophage polarization, thereby establishing an anti-inflammatory environment that protects against colitis and supports recovery after spinal cord injury [14,19]. This phenotypic shift suppresses NF-κB signaling, leading to reduced production of pro-inflammatory cytokines, including TNF-α and IL-6 [19]. Mechanistically, Lac-Phe has been shown to impede the nuclear translocation of the p65 subunit of NF-κB in macrophage models, thereby attenuating M1 polarization [19]. Importantly, the precursor lactate is known to suppress NF-κB activation via the GPR81 (HCAR1) receptor. In contrast, a direct role for Lac-Phe in GPR81-mediated signaling has not been demonstrated. Any potential potentiating or synergistic effect of Lac-Phe on lactate’s anti-inflammatory actions via this pathway remains speculative [20]. At the systemic metabolic level, Lac-Phe enhances glucose homeostasis, potentially via improved mitochondrial function or indirect modulation of insulin signaling, although its direct mechanisms remain unclear [3,9].

As a biomarker, Lac-Phe has been associated with mitochondrial dysfunction [1,3,12]. In MELAS, its levels correlate with disease severity and may offer a diagnostic advantage over lactate, which remains largely unchanged during exertion; however, this advantage has been demonstrated primarily in small-scale metabolomics studies and warrants validation in larger clinical cohorts [1,3,12]. In type 2 diabetes, elevated Lac-Phe associates with insulin resistance, consistent with broader metabolomics evidence highlighting circulating metabolites as indicators and mediators of cardiometabolic dysfunction [2,21]. Lac-Phe has emerged as a potentially sensitive marker of mitochondrial redox imbalance, particularly under conditions of NADH reductive stress, in which an elevated NADH-to-NAD^+^ ratio disrupts electron transport chain function and drives lactate accumulation [1,3,12]. In mitochondrial disorders such as MELAS, N-lactoyl-amino acids, including Lac-Phe, have been observed to accumulate in proportion to disease severity and may outperform lactate as biomarkers due to their stability during physical exertion, although these findings are based on exploratory metabolomics data and require prospective clinical validation [1,11,12]. This elevation likely arises from increased glycolytic flux during reductive stress, in which impaired oxidative phosphorylation leads to substrate overflow, thereby enhancing CNDP2-mediated conjugation of lactate to phenylalanine. In sepsis, circulating N-lactoyl-amino acids, including Lac-Phe, have been shown to correlate with mitochondrial dysfunction and to predict mortality in patients with septic shock, suggesting promising prognostic utility; however, these observations are based on a single cohort study and await independent replication [11,22]. Furthermore, in vitro studies show that exogenous Lac-Phe impairs mitochondrial respiration in hepatic and neuronal models, potentially intensifying oxidative stress by disrupting ATP production and activating inflammatory signaling pathways [9]. Collectively, these findings suggest that Lac-Phe may serve not only as an emerging candidate biomarker but also as a potential regulator of mitochondrial redox homeostasis. However, given that most supporting evidence derives from preclinical models or small-scale metabolomics studies, further mechanistic and clinical investigations are warranted before definitive conclusions about its diagnostic utility can be drawn.

Beyond its metabolic functions, Lac-Phe exerts marked effects, including on NF-κB signaling. In models of colitis and spinal cord injury, Lac-Phe drives macrophage phenotypic switching from the pro-inflammatory M1 state toward the anti-inflammatory M2 state, thereby attenuating reactive oxygen species (ROS) generation and reducing pro-inflammatory cytokines such as TNF-α and IL-6 [14,19]. This polarization is mediated, at least in part, by inhibition of NF-κB activation, as Lac-Phe blocks nuclear translocation of the p65 subunit, thereby dampening ROS-driven inflammatory cascades [19]. Notably, the Lac-Phe precursor lactate suppresses NF-κB through GPR81-dependent mechanisms, suggesting that Lac-Phe may further potentiate lactate-mediated redox modulation to facilitate inflammation resolution [22]. These anti-inflammatory properties underscore the potential of Lac-Phe as a therapeutic agent in conditions characterized by chronic immune activation. Separately, in the context of mitochondrial disease, the association of Lac-Phe with NADH reductive stress reflects a distinct redox-sensing function: elevated Lac-Phe in these states indicates oxidative imbalance and mitochondrial dysfunction rather than an exercise or feeding response [9]. Therapeutic administration of Lac-Phe may also counteract inflammation via the AMPK-PGC1α-PPARγ axis, reducing ROS accumulation in microglia and macrophages [12,14]. These redox-modulatory properties are mechanistically separable from the direct anti-inflammatory effects described above, and together position Lac-Phe as a multifaceted agent operating at the interface of immune regulation and mitochondrial homeostasis.

Exercise, a major inducer of Lac-Phe, engages redox signaling by producing transient oxidative stress that triggers adaptive responses, with Lac-Phe acting as a molecular bridge between glycolytic flux and systemic homeostasis [1,3,13,15]. High-intensity exercise elevates Lac-Phe levels in an intensity-dependent manner, paralleling rises in lactate and other exerkines, such as succinate, which influence mitochondrial signaling and ROS homeostasis [1,23]. This response may support redox adaptations, as lactate-derived metabolites such as Lac-Phe help shuttle reducing equivalents, thereby alleviating exercise-induced oxidative stress and reinforcing anti-inflammatory effects [13,19]. In obese models, exercise-stimulated Lac-Phe correlates with adipose tissue loss and improved insulin sensitivity, potentially by counteracting ROS-driven insulin resistance via NF-κB inhibition and M2 macrophage polarization [2,19]. Collectively, these exercise-related redox findings support a role for Lac-Phe as an endogenous mediator of adaptive responses to physical stress. Pharmacological induction of Lac-Phe by metformin operates through a mechanistically distinct but convergent pathway, the complex I inhibition and reductive stress rather than exercise per se, and is addressed in the context of biosynthetic regulation and therapeutic implications.

Human studies confirm intensity-dependent responses, with high-intensity exercise eliciting greater increases in Lac-Phe than moderate activity, as well as other exerkines, such as succinate [23]. Sex differences are evident, with females exhibiting higher Lac-Phe peaks, which may reflect hormonal influences [24]. Together, these human exercise findings provide direct translational support for the preclinical observations and establish Lac-Phe as a robust, exercise-responsive biomarker in humans. Beyond exercise physiology, emerging, albeit preliminary, evidence suggests additional roles for Lac-Phe in two distinct contexts that warrant separate consideration. In aging, Lac-Phe may counteract age-related anorexia and sarcopenia by transiently suppressing appetite during exercise, supporting longevity without inducing chronic undernutrition [13]. In oncology, early data implicate Lac-Phe in modulating the tumor microenvironment and activating inflammatory pathways through lactate-driven mechanisms [25,26]; however, these observations remain largely mechanistic and require independent validation before clinical implications can be drawn.

Multiple exercise-induced exerkines collectively mediate the systemic physiological benefits of physical activity, including metabolic regulation, anti-inflammatory effects, neuroprotection, and muscle adaptation [27]. However, Lac-Phe stands out for its potent appetite suppression, marked responsiveness to high-intensity exercise (up to a threefold increase), and partial mediation of metformin-induced weight loss [1,2,5,7]. In contrast, other classic and emerging exerkines exhibit distinct emphases. For instance, lactate and succinate increase in concentration in an intensity-dependent manner and function in energy shuttling and mitochondrial signaling [23]. Brain-derived neurotrophic factor (BDNF) is strongly induced by high-intensity exercise, particularly interval training, and promotes neuroplasticity and cognitive performance [28]. Apelin counteracts sarcopenia and enhances muscle regeneration and mitochondrial biogenesis during aging via AMPK and AKT signaling pathways [29]. β-aminoisobutyric acid, a valine-derived metabolite, stimulates white adipose browning, boosts β-oxidation, and supports musculoskeletal health, especially in response to aerobic or endurance exercise [30]. These exerkines often act synergistically with Lac-Phe and other lactate-derived metabolites to orchestrate the broad protective effects of exercise (Table 1). The unique advantages of Lac-Phe in appetite regulation and as a biomarker of mitochondrial dysfunction highlight its potential as a candidate for “exercise-mimicking” therapeutic strategies. Future investigations should explore the interactive networks among these exerkines to optimize personalized exercise prescriptions and pharmacological interventions.

## 4. Therapeutic Implications and Biomarker Potential

The therapeutic promise of Lac-Phe stems from its capacity to partially replicate certain effects of exercise and metformin in preclinical models. In obesity, exogenous Lac-Phe reduces food intake and body weight in rodents, with oral formulations retaining some efficacy despite gut degradation [1]. A phase I trial (NCT06743009) is currently assessing the isolated effects of Lac-Phe on appetite and metabolism in obese individuals using a double-blind crossover design [8].

Important translational challenges include poor oral bioavailability due to susceptibility to gastrointestinal peptidases, which renders intraperitoneal rodent dosing clinically inapplicable, the lack of human pharmacokinetic data, such as plasma half-life, and the need for systematic safety evaluation of potential off-target effects on phenylalanine homeostasis and insulin signaling. Prodrug or stabilized analog strategies may warrant future exploration. In inflammatory contexts, Lac-Phe promotes M2 macrophage polarization, conferring neuroprotection after spinal cord injury and preserving gut barrier integrity in colitis [14,19]. For mitochondrial disorders, Lac-Phe analogs might alleviate redox stress, and their biomarker status could guide personalized therapeutic strategies [1,3,9,12].

## 5. Challenges and Future Directions

Although Lac-Phe shows promise as a signaling metabolite, several important controversies and limitations remain. Most mechanistic evidence derives from rodent models, with human data largely restricted to correlative metabolomics or small-scale studies. The magnitude and consistency of appetite suppression in non-obese humans remain uncertain. The absence of an identified receptor makes many proposed cellular mechanisms hypothetical. Associations with redox homeostasis, mitochondrial dysfunction, and anti-inflammatory effects are primarily correlative rather than proven causal. Lac-Phe elevation occurs not only after exercise but also with feeding and with metformin, which calls into question its strict classification as an “exerkine”. Differences in exercise protocols (intensity, volume, type) and nutritional status further complicate interpretation and translation. Larger, well-controlled human trials, receptor deorphanization, and tissue-specific studies are essential.

Key challenges include identifying the Lac-Phe receptor (likely a G-protein-coupled receptor based on structural homology) and enhancing its bioavailability. Addressing remaining knowledge gaps, particularly receptor identity and tissue-specific contributions, will be essential for clinical translation. Advanced proteomics and CRISPR-based screens may elucidate signaling pathways of Lac-Phe [1,9,24]. Longitudinal human studies are required to evaluate its chronic effects on aging and disease progression [13]. Combining Lac-Phe measurements with multi-omics datasets could enhance their value as biomarkers [1,9,13,14,17,24]. Ethical considerations, including equitable access in therapeutic development, must also be addressed.

## 6. Conclusions

**Figure 1:**
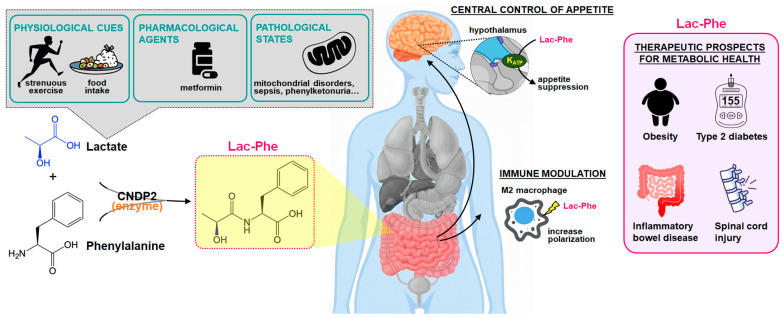
Biosynthesis, physiological regulation, and multifunctional actions of N-lactoyl-phenylalanine (Lac-Phe). Lac-Phe is an N-acyl amino acid signaling metabolite synthesized by carnosine dipeptidase 2 (CNDP2), which conjugates L-lactate and L-phenylalanine via substrate-driven reverse proteolysis in macrophages, monocytes, and intestinal epithelial cells. Three principal stimuli drive biosynthesis: (1) high-intensity exercise, which raises circulating lactate via anaerobic glycolysis; (2) postprandial feeding, which elevates phenylalanine from dietary protein digestion; and (3) metformin, which inhibits mitochondrial complex I, increasing intracellular lactate via lactate dehydrogenase. In pathological states such as MELAS (mitochondrial encephalomyopathy, lactic acidosis, and stroke-like episodes) and septic shock, NADH-reductive stress and impaired oxidative phosphorylation elevate Lac-Phe levels, positioning it as a candidate biomarker of mitochondrial dysfunction. Once in the circulation, Lac-Phe exerts effects across three functional domains: (1) Centrally, it acts on the hypothalamus to suppress orexigenic AgRP neurons while preserving anorexigenic POMC neuronal activity, reducing food intake and promoting weight loss. (2) Peripherally, it promotes M1-to-M2 macrophage polarization, suppressing NF-κB activation and reducing pro-inflammatory cytokines (TNF-α, IL-6), with protective effects in murine models of colitis and spinal cord injury. (3) At the systemic metabolic level, it is associated with improved glucose homeostasis and insulin sensitivity in obese models via mechanisms that remain incompletely understood. Renal excretion via SLC17A1/SLC17A3 maintains systemic homeostasis; CNDP2 loss-of-function models abolish exercise-induced Lac-Phe elevation and attenuate the metabolic benefits of exercise and metformin. Note: arrows represent proposed or partially characterized pathways based on preclinical evidence; the Lac-Phe receptor remains unidentified, and several downstream mechanisms are hypothetical. AgRP, agouti-related peptide; CNDP2, carnosine dipeptidase 2; MELAS, mitochondrial encephalomyopathy, lactic acidosis, and stroke-like episodes; NF-κB, nuclear factor kappa B; POMC, pro-opiomelanocortin; SLC17A1/3, solute carrier family 17 members 1 and 3; TNF-α, tumor necrosis factor-α.

Lac-Phe exemplifies how metabolites can orchestrate complex physiological processes in specific metabolic contexts, ranging from exercise-associated benefits to disease modulation (Figure 1). Continued careful investigation, with attention to the controversies and limitations outlined above, may facilitate the development of novel biomedical interventions.

## Figures and Tables

**Table 1:** Comparison of Lac-Phe with other key exercise-induced exerkines.

Exerkine	Primary Source	Main Physiological Effects	Exercise Intensity Dependence	Key Mechanisms/Targets	References
N-Lactoyl-Phenylalanine (Lac-Phe)	Macrophages, monocytes, epithelial cells (via carnosine dipeptidase 2)	Appetite suppression, weight loss, anti- obesity, improved glucose tolerance, M2 macrophage polarization (anti- inflammatory)	High (vigorous/high-intensity; up to a 3-fold increase)	Hypothalamic AgRP neuron inhibition (via ATP-sensitive potassium channels), NF-κB suppression mediates metformin action	Hoene et al. [2] Li et al. [1] Liu et al. [4] Scott et al. [5] Xiao et al. [7] Yu et al. [19]
Lactate	Skeletal muscle (glycolysis)	Energy shuttle, anti-inflammatory, metabolic signaling, Lac-Phe precursor	High (intensity- dependent surge)	HCAR1 receptor, redox signaling	Li et al. [1] Ying et al. [14] Weber et al. [23] Zhu et al. [31]
Succinate	Skeletal muscle, TCA cycle	Inflammatory modulation, mitochondrial signaling	High (intensity- dependent)	SUCNR1 receptor, pro-/anti- inflammatory balance	Weber et al. [23]
Irisin	Skeletal muscle (FNDC5 cleavage)	White adipose tissue browning, thermogenesis, insulin sensitivity, anti-inflammation	Moderate to high (acute; high- intensity interval training/endurance)	αVβ5 integrins, PGC-1α pathway	Guo et al. [32] He et al. [33] Thrones et al. [34]
Fibroblast growth factor 21 (FGF21)	Liver, skeletal muscle, white adipose tissue	Glucose/lipid metabolism, energy expenditure, anti- obesity	Variable (time- dependent post- exercise)	FGFR1/KLB complex, ERK1/2 pathway	Thrones et al. [34] Xu et al. [35]
IL-6	Skeletal muscle (contraction-induced)	Acute: lipolysis, glucose uptake; chronic: anti- inflammation	High prolonged/endurance	IL-6R signaling, AMPK activation, GLUT4 translocation	Ikeda et al. [36] University of Aarhus. [8] Thrones et al. [34] Yu et al. [19]
Brain-derived neurotrophic factor (BDNF)	Skeletal muscle, brain	Neuroprotection, synaptic plasticity, cognition, muscle metabolism, anti- inflammation	High intensity (acute exercise/high-intensity interval training)	TrkB receptor, CaMKII/AKT signaling, PGC-1α, blood–brain barrier penetration	Cheng et al. [37] Cheng et al. [38] Mielniczek et al. [28]
Apelin	Skeletal muscle, adipose tissue	Muscle homeostasis, mitochondrial biogenesis, anti- sarcopenia, glucose uptake, insulin sensitivity, anti- inflammation	Moderate to high intensity (high- intensity interval training/endurance; declines with aging)	APLNR receptor, AMPK/AKT/mTOR signaling, satellite cell activation	Kilpiö et al. [29] Thrones et al. [34]
L-β- aminoisobutyric acid (L-BAIBA)	Skeletal muscle (valine catabolism)	White adipose tissue browning, β- oxidation, bone protection, improved insulin sensitivity, musculoskeletal enhancement in aging	Moderate to high intensity (aerobic/endurance)	MRGPRD receptor, AMPK/PI3K/Akt signaling, PGC-1α- dependent	Huang et al. [39] Kitase et al. [40] Vallejo et al. [30]

Note: Lactate is included as a key metabolic precursor and signaling molecule rather than a canonical exerkine. IL-6 exerts both pro-inflammatory and anti-inflammatory effects depending on context and chronicity. The human physiological relevance of irisin remains controversial owing to historical challenges in detection. Levels of evidence vary, with many findings stronger in rodent models than in humans.

## Data Availability

No new data were created or analyzed in this study. Data sharing is not applicable to this article.
